# The association of genetic variation in CACNA1C with resting-state functional connectivity in youth bipolar disorder

**DOI:** 10.1186/s40345-022-00281-5

**Published:** 2023-01-13

**Authors:** Xinyue Jiang, Alysha A. Sultan, Mikaela K. Dimick, Clement C. Zai, James L. Kennedy, Bradley J. MacIntosh, Benjamin I. Goldstein

**Affiliations:** 1 Centre for Youth Bipolar Disorder, Centre for Addiction and Mental Health, Toronto, ON Canada; 2 Department of Pharmacology & Toxicology, University of Toronto, Toronto, ON Canada; 3 Tanenbaum Centre for Pharmacogenetics, Psychiatric Neurogenetics Section, Campbell Family Mental Health Research Institute, Centre for Addiction and Mental Health, Toronto, ON Canada; 4 Department of Psychiatry, University of Toronto, Toronto, Canada; 5 Heart and Stroke Foundation, Canadian Partnership for Stroke Recovery, Sunnybrook Research Institute, Toronto, ON Canada; 6 Department of Medical Biophysics, University of Toronto, Toronto, ON Canada; 7 Hurvitz Brain Sciences Program, Sunnybrook Research Institute, Toronto, ON Canada

**Keywords:** Bipolar disorder, Youth, fMRI, Resting-state functional connectivity, *CACNA1C* rs1006737, Seed-to-voxel analysis, Anterior cingulate cortex, Orbitofrontal Cortex, Amygdala

## Abstract

**Background:**

*CACNA1C* rs1006737 A allele, identified as a genetic risk variant for bipolar disorder (BD), is associated with anomalous functional connectivity in adults with and without BD. Studies have yet to investigate the association of *CACNA1C* rs1006737 with resting-state functional connectivity (rsFC) in youth BD.

**Methods:**

Participants included 139 youth with BD-I, -II, or -not otherwise specified, ages 13–20 years, including 27 BD A-carriers, 41 BD non-carriers, 32 healthy controls (HC) A-carriers, and 39 HC non-carriers. Anterior cingulate cortex (ACC), amygdala, and orbitofrontal cortex (OFC) were examined as regions-of-interest in seed-to-voxel analyses. General linear models included main effects of diagnosis and rs1006737, and an interaction term, controlling for age, sex, and race.

**Results:**

We observed a main effect of BD diagnosis on rsFC between the right amygdala and the right occipital pole (*p* = 0.02), and a main effect of rs1006737 genotypes on rsFC between the right OFC and bilateral occipital cortex (*p* < 0.001). Two significant BD diagnosis-by-CACNA1C rs1006737 interactions were also identified. The A allele was associated with positive rsFC between the right ACC and right amygdala in BD but negative rsFC in HC (*p* = 0.01), and negative rsFC between the left OFC and left putamen in BD but positive rsFC in HC (*p* = 0.01).

**Conclusion:**

This study found that the rs1006737 A allele, identified as a genetic risk variant for BD in adults, was differentially associated with rsFC in youth with BD in regions relevant to emotion, executive function, and reward. Future task-based approaches are warranted to better understand brain connectivity in relation to *CACNA1C* in BD.

**Supplementary Information:**

The online version contains supplementary material available at 10.1186/s40345-022-00281-5.

## Introduction

Bipolar disorder (BD) is a severe recurrent mood disorder, characterized by episodes of mania and/or hypomania and depression, and affects 2–5% of adolescents worldwide (Goldstein et al. 2017; Van Meter et al. 2011). Large-scale genome-wide association studies (GWAS) have consistently identified several risk-conferring genetic variants for adult BD, including the single nucleotide polymorphism (SNP) rs1006737 at the calcium channel, voltage-dependent, L-type, alpha-1C subunit (*CACNA1C*) gene locus (Ferreira et al. 2008; Psychiatric GWAS Consortium Bipolar Disorder Working Group 2011). The *CACNA1C* gene encodes for the alpha subunit of the Cav1.2 L-type voltage-dependent calcium channel, which regulates neuronal plasticity and may have direct effects on transcription of genes involved in neuronal signaling and excitability (Gomez-Ospina et al. 2006; Moon et al. 2018). This subunit also contributes to the development and maturation of parvalbumin (PV) γ-aminobutyric acid–transmitting (GABAergic) interneurons (Jiang and Swann 2005).

While *CACNA1C* rs1006737 is identified as a credible susceptibility locus for BD, the neural mechanism of such association is still unclear. The rs1006737 risk A allele has been associated with anomalous activation in several brain regions within the frontotemporal circuit in task-based fMRI studies of adults with and without BD (Ou et al. 2015). One study of healthy adolescents found that, compared to G allele carriers, A allele homozygotes exhibited increased amygdala activation when viewing negative stimuli and decreased amygdala activation when instructed to downregulate their emotional response to negative stimuli (Sumner et al. 2015). In addition to regional task-related brain activation, rs1006737 has been associated with aberrant functional connectivity in both adults with and without BD (Janiri et al. 2021; Ou et al. 2015). Task-based functional connectivity studies of healthy adults have found that rs1006737 A allele carriers showed altered fronto-limbic connectivity, including amygdala, prefrontal cortex, hippocampus, medial temporal lobe, and anterior cingulate cortex (ACC) (Cosgrove et al. 2017; Erk et al. 2010; Paulus et al. 2014; Wang et al. 2011). In adults with BD, rs1006737 risk allele was found to be associated with reduced visual-prefrontal effective connectivity (Dima et al. 2013), as well as decreased effective connectivity from medial frontal gyrus (Radua et al. 2013) during emotion processing tasks.

Thus far, no studies have examined rs1006737 genotypes in relation to functional connectivity in youth with BD. Similar to adults, studies of youth with BD have consistently found altered functional connectivity during resting state (Cattarinussi et al. 2022; Dickstein et al. 2010; Gao et al. 2014; Singh et al. 2015; Stoddard et al. 2015; Xiao et al. 2013), as well as during affective and cognitive tasks (Chang et al. 2017; Passarotti et al. 2012; Rich et al. 2008; Ross et al. 2021; Wang et al. 2012; Wegbreit et al. 2011). Particularly, amygdala abnormalities are amongst the most consistent findings in youth with BD. Furthermore, a previous study from our group found significant BD-by-rs1006737 interactions on the ACC and prefrontal regions including orbitofrontal cortex (OFC) such that BD A-carriers had significantly larger volume and/or surface area relative to both BD non-carriers and healthy A-carriers (Shonibare et al. 2021). We therefore examined rs1006737 genotypes in relation to resting-state functional connectivity (rsFC) in youth BD in three regions of interest (ROIs): ACC, amygdala, and OFC. We hypothesized that the association of the risk A allele with rsFC patterns in the chosen ROIs would differ in youth with BD when compared to the healthy controls (HC).

## Methods

### Participants

This study included 139 youth, ages 13–20 years old. BD participants (type I, II, or not otherwise specified [NOS]) were recruited from a subspecialty youth BD clinic at an academic health sciences centre in Toronto, Ontario, Canada; HC participants were recruited from the community through advertisements. HC participants had no history of major mood diagnoses, recent anxiety disorders, or any first- or second-degree relative with BD or psychotic disorders. Exclusion criteria were: (1) unable to provide informed consent; (2) pre-existing cardiac, auto-immune, or inflammatory conditions; (3) currently taking any anti-inflammatory, anti-platelet, anti-lipidemic, anti-hypertensive, or hypoglycemic agents; (4) any infectious illness within the 14 days prior to the study; (5) any MRI contraindications (i.e. any metal in the body, claustrophobia, etc.); (6) any severe neurological or cognitive impairments; (7) substance dependence in the past 3 months. Written informed consent was obtained from all participants, as well as their parent(s) or guardian(s). Ethical approval was granted by Sunnybrook Research Institute Research Ethics Board. All data was collected at Sunnybrook Research Institute, and was transferred with the Centre for Youth Bipolar Disorder’s relocation to the Centre for Addiction and Mental Health. Ethical approval was also granted by CAMH Research Ethics Board.

### Diagnostic interview & symptom ratings

All interviews were performed by trained study personnel with either bachelor’s or master’s degree in a health-related field and completed comprehensive Schedule for Affective Disorders and Schizophrenia for School-Age Children, Present and Lifetime version (K-SADS-PL) training under the supervision of the senior author (B.I.G.), a licensed child-adolescent psychiatrist. Diagnostic and symptom ratings were reviewed and confirmed by a licensed child and adolescent psychiatrist. K-SADS-PL (Kaufman et al. 1997), a semi-structured interview with both parent and youth, was used to assess current and lifetime psychiatric disorders for all participants. BD subtypes I and II were defined using the DSM-IV criteria, as participants were enrolled from 2014 to 2019, and the DMS-5 version of K-SADS-PL was not available until 2016. BD-NOS was defined using operationalized criteria as per the Course and Outcome of Bipolar Youth study (Birmaher et al. 2006). K-SADS Mania Rating Scale and K-SADS Depression Rating Scale were used to determine mood symptoms of BD participants (Axelson et al. 2003; Chambers et al. 1985). Details regarding clinical measures are described in the Additional file 1.

### Genotyping

Methods of saliva and DNA extraction can be found in Additional file 1. The *CACNA1C* rs1006737 SNP was genotyped using a TaqMan^®^ genotyping assay (C___2584015_10) following manufacturer procedures and analyzed on the QuantStudio 12 K Flex PCR System (Thermo Fisher Scientific, Burlington, ON, Canada). Genotyping of 10% of samples from each run was replicated for quality control purposes for each marker. All genetic sample processing (DNA extraction and genotyping) was performed by the CAMH Biobank and Molecular Core Facility. Technicians were blinded to diagnosis. Participants homozygous for the G-allele (GG) were defined as the rs1006737 non-carrier group, while participants who were either heterozygous or homozygous for the A-allele (AG/AA) were defined as the risk allele-carrier group/A-carriers. Hardy–Weinberg Equilibrium was performed using PLINK software version 1.90, and the genotypes were within Hardy–Weinberg equilibrium (p > 0.05) in the overall sample, as well as in the BD and HC group (Hosking et al. 2004; Purcell et al. 2007).

### Magnetic resonance imaging acquisition

Neuroimaging was acquired using a 3 Tesla (3 T) Philips Achieva system with an 8-channel head receiver coil and body coil transmission. Structural imaging was acquired using T1-weighted high-resolution fast-echo imaging (repetition time (TR)/echo time (TE)/inversion time (TI) = 9.5/2.3/1400 ms, spatial resolution = 0.94 × 1.17 × 1.2 mm, 256 × 164 × 140 matrix, scan duration = 8 m 56 s, 140 slices). Resting-state fMRI was acquired using T2^*^-weighted echo planar imaging (TR/TE = 1500/30 ms, flip angle = 70°, ascending slices, field of view (FOV) = 230 × 181 mm, spatial resolution = 3 × 3 × 4 mm, matrix 76 × 60 × 28, volumes = 230). During the resting-state scan, participants were instructed to keep their eyes open, focus on a blank screen, and not think about anything in particular.

### Functional magnetic resonance imaging analysis

Processing steps and subsequent analyses were completed using the CONN toolbox (v17) in Matlab and SPM12 (Van Dijk et al. 2010; Whitfield-Gabrieli and Nieto-Castanon 2012). The first three volumes of the functional data were removed to account for signal equilibration. Data preprocessing of functional volumes was performed using the default pipeline for volume-based analyses in the CONN toolbox. The pipeline included functional realignment and unwarping (participant motion estimation and correction), alignment of functional data to structural images, slice-timing correction, functional outlier detection (Artifact Detection Tools (ART)-based identification of outlier scans for scrubbing), segmentation of brain tissue and volumes based on Montreal Neurological Institute image reference coordinates (i.e. gray/white/cerebrospinal fluid segmentation), as well as functional smoothing (8 mm FWHM Gaussian filter). Problematic time points were identified using ART within the CONN toolbox to account for head motion. Images were defined as outliers if the global mean intensity of the image was > 3 standard deviations from mean image intensity for the entire resting scan, alternatively if there was a displacement of > 1.0 mm from previous frame in one of the x, y, or z direction. Moreover, volumes were manually examined for motion outliers (> 2 mm or 2 degree rotation in any direction: x, y, z), and participants were excluded if they had any volumes with a problematic level of motion outliers (i.e. 24 participants were excluded: 16 BD and 8 HC). Denoising was performed using CONN’s default pipeline, which combined a linear regression of potential confounds in the blood oxygen level dependent (BOLD) signal (including white matter, cerebrospinal fluid, re-alignment, scrubbing, and effect of rest), and band-pass filtering (0.008–0.09 Hz). Two independent raters examined the histograms from the functional connectivity values for each participant after denoising, and revealed normally distributed data for all participants not previously excluded due to head motion.

Seed selection was determined a priori and included the ACC, amygdala, and OFC, which were identified using the FMRIB Software Library (FSL) Harvard–Oxford structural atlas generated by the CONN toolbox. All seeds were parcellated into left and right hemisphere within the atlases.

### Statistical analysis

SPSS Version 27 was used to perform all statistical analyses. Demographic characteristics were compared among groups (BD A-carriers, BD non-carriers, HC A-carrier, and HC non-carriers) using analysis of variance for continuous variables, and chi-square tests for categorical variables. Clinical characteristics were compared between the BD A-carriers and BD non-carriers using t-tests for continuous variables and chi-squared tests for categorical variables. Non-parametric tests (Mann–Whitney *U*-tests, Kruskal–Wallis) were used for variables that were not normally distributed. Statistical significance was set at *p* < 0.05.

A seed-to-voxel approach was used for functional connectivity analyses. Whole-brain connectivity maps were created by computing Fischer-transformed bivariate correlation coefficients between the time series for each bilateral seed region and all other voxel BOLD time series. Beta values represent Fischer-transformed correlation coefficient values. A general linear model (GLM) was used to determine the group-wise main effects of BD diagnosis and *CACNA1C* rs1006737, as well as group by gene interaction effects. Demeaned age, sex, and race were included as covariates. Voxel-wise height threshold was set at *p* < 0.001 false-discovery rate (FDR) corrected, and cluster thresholding was set at *p* < 0.05 FDR corrected. A Bonferroni correction for multiple comparisons was implemented to account for the three seed-to-voxel contrasts (i.e. *p* < 0.017). Significant clusters from the GLM analyses were exported as masks to conduct post-hoc pairwise comparisons in ROI-to-ROI analyses.

## Results

### Demographic and clinical characteristics

A total of 139 youths were included in analyses: 27 BD A-carriers, 41 BD non-carriers, 32 HC A-carriers, and 39 HC non-carriers. Demographic and clinical characteristics are presented in Table 1. Age (*p* = 0.01), race (*p* = 0.01), and BMI (*p* = 0.01) were significantly different among the groups. The groups did not significantly differ in sex ratios. Compared to the BD non-carrier group, the BD A-carrier group had significantly higher current mania score (*p* = 0.01) and higher proportion of participants currently taking non-SSRI antidepressants (*p* = 0.01).

**Table 1:** Demographic and clinical characteristics

	BD		HC		Test statistic	*p*-value	Effect size
	A-carrier (n = 27)	Non-carrier (n = 41)	A-carrier (n = 32)	Non-carrier (n = 39)			
Age, years	17.4 ± 1.8	17.5 ± 1.4	16.2 ± 1.6	17.1 ± 1.7	F = 4.46	**0.01**^df^	η^2^ = 0.09
Sex (n, % female)	18 (66.7)	25 (61.0)	17 (53.1)	22 (56.4)	χ2 = 1.29	0.73	V = 0.10
SES	4.3 ± 0.9	4.3 ± 1.0	4.4 ± 0.8	4.3 ± 1.0	H = 0.82	0.85	η^2^ = 0.02
Race (n, % caucasian)	22 (81.5)	28 (68.3)	23 (71.9)	17 (43.6)	χ2 = 11.90	**0.01**^bce^	V = 0.29
Intact family (n, %)	17 (63.0)	24 (58.5)	23 (71.9)	23 (59.0)	χ2 = 1.69	0.64	V = 0.11
Tanner stage (1–5)	4.4 ± 0.7	4.4 ± 0.6	4.3 ± 0.7	4.2 ± 0.6	H = 4.94	0.18	η^2^ = 0.02
BMI (adjusted)	24.0 ± 5.2	24.0 ± 4.2	22.0 ± 4.5	21.5 ± 2.9	H = 11.62	**0.01**^e^	η^2^ = 0.06
Clinical characteristics							
BD-I	9 (33.3)	14 (34.1)			χ^2^ = 0.24		
BD-II	8 (29.6)	14 (34.1)				0.89	V = 0.06
BD-NOS	10 (37.0)	13 (31.7)					
Age of BD onset	15.17 ± 1.92	14.47 ± 3.14			U = 585.0	0.69	d = 0.26
Lifetime psychosis	6 (22.2)	13 (31.7)			χ^2^ = 0.71	0.39	V = 0.10
Lifetime suicide attempts	4 (14.8)	6 (14.6)			χ^2^ = 0.0	0.98	V = 0.002
Lifetime self-injurious behaviour	17 (63.0)	21 (51.2)			χ^2^ = 0.91	0.34	V = 0.12
Lifetime suicidal ideation	21 (77.8)	23 (56.1)			χ^2^ = 3.35	0.07	V = 0.22
Police contact/arrest	3 (11.1)	13 (31.7)			χ^2^ = 3.84	0.05	V = 0.24
Lifetime physical abuse	0	2 (6.3)			χ^2^ = 1.30	0.25	V = 0.16
Lifetime sexual abuse	0	3 (9.4)			χ^2^ = 2.0	0.16	V = 1.0
Lifetime psychiatric hospitalization	10 (37.0)	22 (53.7)			χ^2^ = 1.81	0.18	V = 0.16
Current depression score	18.0 ± 10.64	13.85 ± 10.59			U = 691.50	0.08	d = 0.39
Lifetime depression score	32.0 ± 12.84	28.85 ± 10.12			t = − 1.13	0.26	d = 0.28
Current mania score	13.11 ± 10.06	7.10 ± 8.78			U = 755.0	**0.01**	d = 0.64
Lifetime mania score	31.59 ± 9.30	29.56 ± 10.48			t = − 0.82	0.42	d = 0.20
CGAS—most severe past episode	44.54 ± 8.61	43.44 ± 9.27			U = 584.50	0.50	d = 0.12
CGAS—highest past year	68.81 ± 10.66	67.59 ± 12.42			U = 566.50	0.66	d = 0.10
CGAS—past month	65.27 ± 11.11	64.80 ± 12.11			U = 557.0	0.76	d = 0.04
Lifetime comorbid diagnoses							
ADHD	15 (55.6)	19 (46.3)			χ^2^ = 0.55	0.46	V = 0.09
Any anxiety	23 (85.2)	32 (78.0)			χ^2^ = 0.54	0.47	V = 0.09
SUD	6 (30.0)	8 (29.6%)			χ^2^ = 0.001	0.98	V = 0.004
ODD	6 (22.2%)	13 (31.7)			χ^2^ = 0.73	0.39	V = 0.10
CD	0	2 (4.9)			χ^2^ = 1.36	0.24	V = 0.14
Nicotine use (yes/no)	11 (40.7)	18 (43.9)			χ^2^ = 0.07	0.80	V = 0.03
Alcohol abuse	3 (11.1)	2 (4.9)			χ^2^ = 0.93	0.34	V = 0.12
Alcohol dependence	2 (7.4)	2 (4.9)			χ^2^ = 0.19	0.66	V = 0.05
Family psychiatric history							
Mania/hypomania	12 (48.0)	27 (69.2)			χ^2^ = 2.89	0.09	V = 0.21
Depression	19 (76.0)	32 (82.1)			χ^2^ = 0.35	0.56	V = 0.07
Suicide attempt	7 (28.0)	18 (46.2)			χ^2^ = 2.11	0.15	V = 0.18
Anxiety	20 (80.0)	26 (66.7)			χ^2^ = 1.34	0.25	V = 0.15
ADHD	8 (32.0)	15 (38.5)			χ^2^ = 0.28	0.60	V = 0.07
Lifetime medications							
SGA	22 (81.5)	26 (63.4)			χ^2^ = 2.56	0.11	V = 0.19
Lithium	8 (29.6)	8 (19.5)			χ^2^ = 0.93	0.34	V = 0.12
SSRI antidepressants	10 (37)	15 (36.6)			χ^2^ = 0.001	0.97	V = 0.005
Non-SSRI antidepressants	6 (22.2)	7 (17.1)			χ^2^ = 0.28	0.60	V = 0.06
Stimulants	8 (29.6)	9 (22.0)			χ^2^ = 0.51	0.47	V = 0.09
Valproate	2 (7.4)	3 (7.3)			χ^2^ = 0.0	1.0	V = 0.002
Lamotrigine	5 (18.5)	10 (24.4)			χ^2^ = 0.33	0.57	V = 0.07
Any medications	25 (92.6)	32 (78.0)			χ^2^ = 2.54	0.11	V = 0.19
Current medications							
SGA	19 (70.4)	26 (53.4)			χ^2^ = 0.35	0.55	V = 0.07
Lithium	7 (25.9)	7 (17.1)			χ^2^ = 0.78	0.38	V = 0.11
SSRI antidepressants	2 (7.4)	3 (7.3)			χ^2^ = 0.0	0.99	V = 0.002
Non-SSRI antidepressants	4 (14.8)	0			χ^2^ = 6.45	**0.01**	V = 0.31
Stimulants	3 (11.1)	3 (7.3)			χ^2^ = 0.29	0.59	V = 0.07
Valproate	0	0			–	–	–
Lamotrigine	4 (14.8)	11 (26.8)			χ^2^ = 1.37	0.24	V = 0.14

Values are reported in mean ± standard error unless otherwise indicated. Values in bold indicate *p* values at α = 0.05.

*BD* Bipolar Disorder, *HC* Healthy Controls, *SES* Socio-Economic Status, *BMI* body mass index, *NOS* Not Otherwise Specified; Depression Score Based on Depression Rating Scale; Mania Score Based on Mania Rating Scale; *CGAS* Children’s Global Assessment Scale, *ADHD* Attention Deficit-Hyperactivity Disorder, *SUD* Substance Use Disorder, *ODD* Oppositional Defiant Disorder, *CD* Conduct Disorder, *SGA* Second Generation Antipsychotic, *SSRI* Selective Serotonin Reuptake Inhibitor. Post-hpc pairwise comparisons:

^a^BD A-carrier vs. BD non-carrier, p < 0.05

^b^HC A-carrier vs. HC non-carrier, p < 0.05

^c^BD A-carrier vs HC non-carrier, p < 0.05

^d^BD non-carrier vs HC A-carrier, p < 0.05

^e^BD non-carrier vs HC non-carrier, p < 0.05

^f^BD A-carrier vs HC A-carrier, p < 0.05

### Main effects of BD diagnosis and CACNA1C rs1006737

**Fig. 1:**
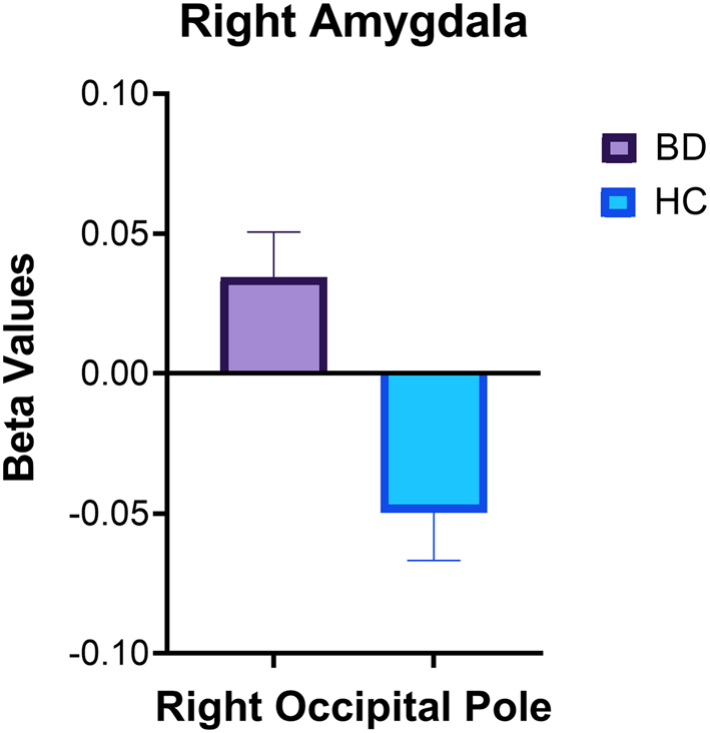
The main effect of bipolar disorder (BD) diagnosis on rsFC between the right amygdala seed and right occipital pole (*p* = 0.02). Beta values correspond to Fischer-transformed correlation coefficient values, adjusted for age, sex, and race. Error bars denote standard error of the mean. *HC* healthy controls

**Fig. 2:**
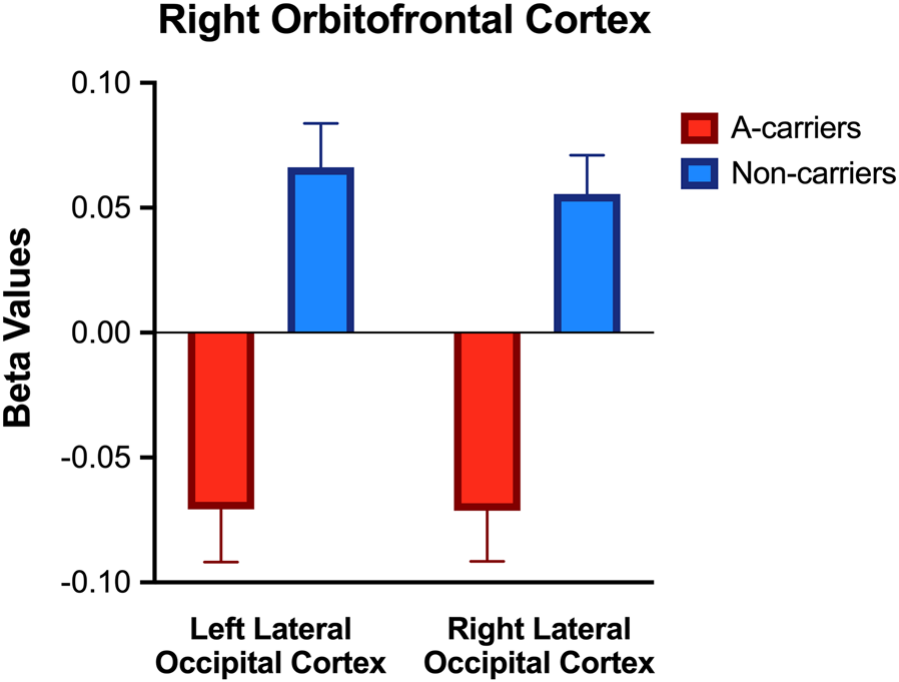
The main effect of *CACNA1C* rs100737 on rsFC between the right orbitofrontal cortex seed and bilateral occipital cortex (*p* < 0.001). Beta values correspond to Fischer-transformed correlation coefficient values, adjusted for age, sex, and race. Error bars denote standard error of the mean

The main effects of BD diagnosis and rs1006737 genotypes on rsFC are listed in Table 2. A significant main effect of BD diagnosis was identified between the right amygdala seed and the right occipital pole, with the between-group difference explained by positive connectivity in the BD group and negative connectivity in the HC group (*p* = 0.02; Fig. 1). However, this finding did not survive the multiple comparison criteria (*p* < 0.017). Additionally, a significant main effect of rs1006737 was revealed between the right OFC seed and the bilateral occipital cortex, with the between-group difference explained by negative connectivity in the A-carrier group and positive connectivity in the non-carrier group (*p* < 0.001; Fig. 2).

**Table 2:** Characteristics of significant rsFC clusters

Seeds	MNI coordinates			Cluster size	Size p-corrected (FDR)	Main region	Additional region(s)
	x	y	z				
Main effect of BD diagnosis							
Right amygdala	+ 18	− 96	+ 24	136	0.02^a^	Right occipital pole	Right intracalcarine cortex, right lateral occipital cortex, right cuneal cortex
Main effect of *CACNA1C* rs1006737							
Right OFC	− 26	− 90	+ 24	382	< 0.001	Left lateral occipital cortex	Left occipital pole
	+ 42	− 78	+ 14	376	< 0.001	Right lateral occipital cortex	Right occipital pole
BD diagnosis x *CACNA1C* rs1006737 interaction effect							
Right ACC	+ 08	− 04	− 20	161	0.01	Right amygdala	Right hippocampus
	− 32	− 08	+ 12	107	0.03^a^	Left insular cortex	Left putamen, left central operculum
Left OFC	− 24	− 06	+ 08	153	0.01	Left putamen	Left caudate, left pallidum

*BD* Bipolar Disorder, *MNI* Montreal Neurological Institute, *FDR* False Discovery Rate, *ACC* anterior cingulate cortex, *OFC* orbital frontal cortex

^a^Did not survive Bonferroni multiple comparisons (p < 0.017)

### BD diagnosis x CACNA1C rs1006737 interaction effects

**Fig. 3:**
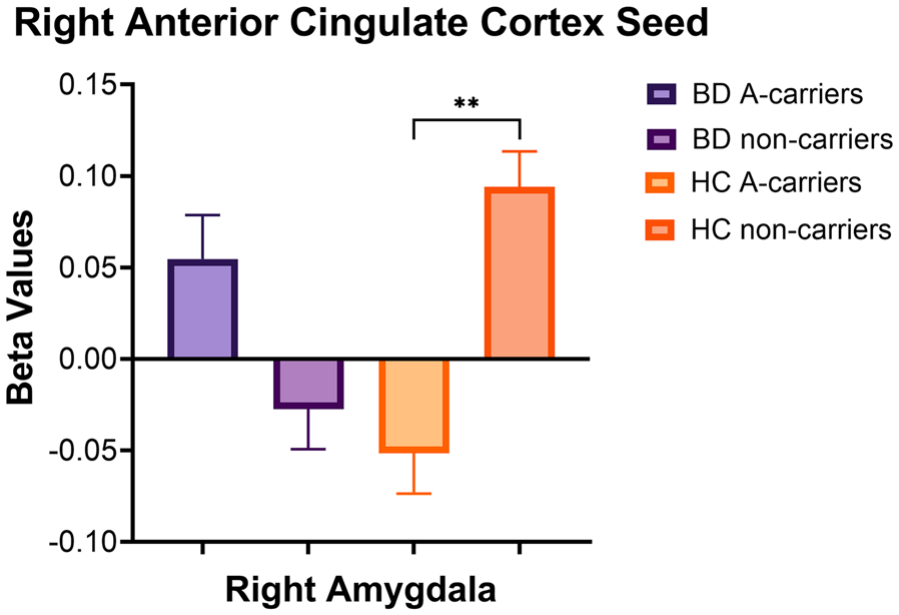
Interaction between bipolar disorder (BD) diagnosis and *CACNA1C* rs1006737 on rsFC between the right anterior cingulate cortex seed and right amygdala. Beta values correspond to Fischer-transformed correlation coefficient values, adjusted for age, sex, and race. Error bars denote standard error of the mean. *HC* healthy controls. **p < 0.01

The BD diagnosis-by-rs1006737 interactions on rsFC are listed in Table 2. Significant differences in rsFC were observed between the right ACC seed and two clusters, including the right amygdala (*p* = 0.01) as well as the left insular cortex *(p* = 0.03). After correction for multiple comparisons, only the right amygdala cluster remained significant. From a visual perspective, Fig. 3 suggests that the significant interaction for the right amygdala cluster is related to positive connectivity in BD A-carriers but negative connectivity in HC A-carriers. However, in post-hoc testing, the only significant pairwise comparison was between HC A-carriers (negative rsFC) and HC non-carriers (positive rsFC; *p* = 0.01).

**Fig. 4:**
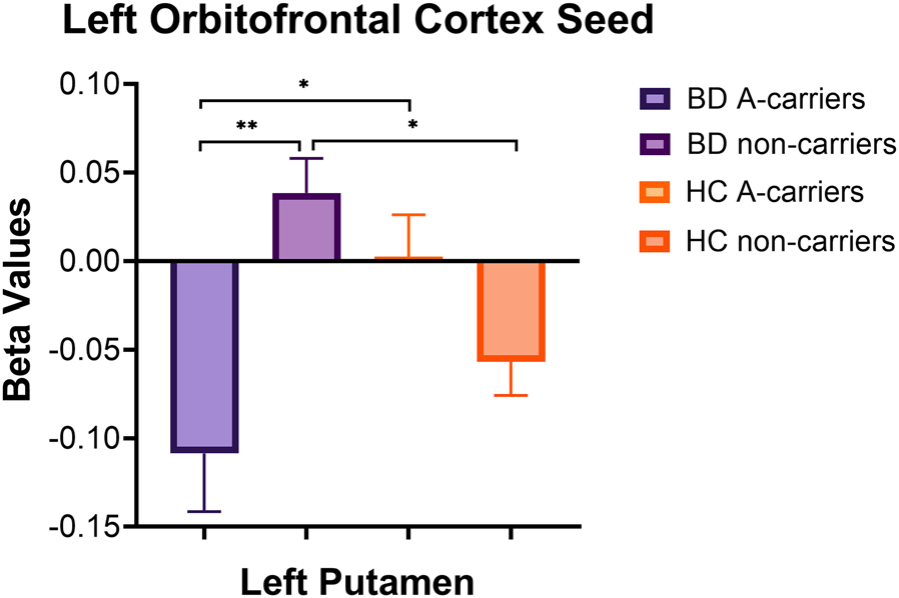
Interaction between bipolar disorder (BD) diagnosis and *CACNA1C* rs1006737 on rsFC between left orbitofrontal cortex seed and left putamen. Beta values correspond to Fischer-transformed correlation coefficient values, adjusted for age, sex, and race. Error bars denote standard error of the mean. *HC* healthy controls. *p < 0.05, **p < 0.01

A significant BD diagnosis-by- rs100673interaction was also observed between the left OFC seed and left putamen (*p* = 0.01), which survived correction for multiple comparisons. Pairwise post-hoc analysis showed significant anti-correlation in BD A-carriers compared to BD non-carriers (*p* = 0.004) and HC A-carriers (*p* = 0.04), such that BD A-carriers had negative connectivity while BD non-carriers and HC A-carriers had positive connectivity. In addition, there was a significant anti-correlation between BD non-carriers and HC non-carriers, such that BD non-carriers had positive connectivity while HC non-carriers had negative connectivity (*p* = 0.03). See Fig. 4 for left OFC pairwise post-hoc results.

## Discussion

The current study bridges a gap in knowledge regarding the association between *CACNA1C* rs1006737 and rsFC in youth with BD. We found a main effect of BD diagnosis on rsFC between the right amygdala seed and the right occipital pole, which did not survive correction for multiple comparisons. In addition, there was a main effect of *CACNA1C* rs1006737 on rsFC between the right OFC seed and bilateral occipital cortex. Finally, there were significant BD diagnosis-by-rs1006737 interactions on rsFC between the right ACC seed and right amygdala, as well as between the left OFC and left putamen, such that rs1006737 risk A allele appeared to have opposite effects on rsFC in the BD group relative to the HC group. This study adds to the literature regarding neurofunctional intermediate phenotypes associated with rs1006737 in BD, extending the study of this topic to include a youth sample earlier in their course of illness. While rs1006737 was identified as a risk factor for BD in studies of adults, present findings demonstrate that this genetic marker is also associated with differential functional connectivity among youth with BD.

### Main effects of BD diagnosis and CACNA1C rs1006737 genotype

We found that the BD group demonstrated positive connectivity between the right amygdala seed and the right occipital pole, while the HC group showed negative connectivity, although the difference did not survive correction for multiple comparisons. In addition to the effect of BD diagnosis, the A-carrier group exhibited negative connectivity between right OFC and left and right lateral occipital cortex, while the non-carrier group had positive connectivity. Amygdala and OFC are two key regions involved in emotion processing and regulation (Anderson 2007; Rolls et al. 2020), while the occipital lobe has been implicated in facial recognition and processing (Grill-Spector et al. 2001; Rehman and Al Khalili 2021). A previous study of adults suggested that BD diagnosis and rs1006737 genotype are both independently linked to dysfunction in the facial-emotional processing network (Dima et al. 2013). Present findings further reflect that, in a population of youth, rs1006737 genotype may be associated with rsFC between occipital regions and OFC, which plausibly represents the neural underpinning of abnormalities in facial affective processing and recognition that occur among individuals with BD (Furlong et al. 2021).

### BD diagnosis x CACNA1C rs1006737 interaction effects

Present findings indicate negative rsFC between the right ACC seed and right amygdala in HC A-carriers relative to HC non-carriers, who demonstrated positive connectivity. A systematic review found that reduced ACC activity but increased amygdala activity among healthy A-carriers compared to healthy non-carriers has been reported in a number of studies of adults (Ou et al. 2015). This may correspond to the negative connectivity between right ACC and right amygdala in HC A-carriers in the current study.

While the only significant pairwise difference was between HC A-carriers and HC non-carriers, a pattern of BD diagnosis-by-genotype interaction emerged that rs1006737 risk A allele was associated with positive rsFC in the BD group but negative rsFC in the HC group. In a prior neurostructural study, our group reported significant BD diagnosis-by-rs1006737 interactions on the ACC structure, such that BD A-carriers had greater volume and surface area relative to BD non-carriers and HC A-carriers^28^. Future studies are warranted to evaluate the nature of the associations between brain structure and rsFC among youth with BD, particularly in relation to rs1006737. With extensive connection to both frontal cortex and the limbic system, ACC is thought to play an important role in the integration of neuronal circuitry for emotion regulation (Stevens et al. 2011). The current finding suggests that *CACNA1C* rs1006737 may be implicated in BD-related emotional dysregulation through differential effects on rsFC within key emotion-regulating regions.

A significant BD diagnosis-by-genotype interaction was also found on rsFC between left OFC and left putamen, such that rs1006737 risk A allele appeared to associate with negative connectivity in the BD group but positive connectivity in the HC group. Structural connectivity and rsFC between OFC and putamen was previously reported in healthy adults, and is thought to have a role in integrating reward and executive processes (Jarbo and Verstynen 2015). A previous study of adults reported a similar pattern of interaction on putamen structure, such that the A allele was associated with smaller putamen volume in the BD group, but increased putamen volume in the HC group (Perrier et al. 2011). Again, future studies regarding the interrelationships between structure and function are warranted.

### Putative neural mechanisms underlying present CACNA1C rs1006737 findings

The role of the rs1006737 continues to be an active area of neuroscience research. rs1006737 risk A allele has been associated with altered *CACNA1C* messenger RNA levels in a region-specific and neuronal subtype-specific manner (Bigos et al. 2010; Eckart et al. 2016; Gershon et al. 2014; Roussos et al. 2014; Tecelão et al. 2019; Wang et al. 2021; Yoshimizu et al. 2015), which may subsequently result in destabilization of calcium-dependent pathways essential for synaptic plasticity, learning, and memory process (Balog et al. 2010; Gershon et al. 2014; Moon et al. 2018; Roussos et al. 2014). Additionally, it has been proposed that the hyperactive glutamatergic pathway in BD may generate higher probabilities for abnormalities in the excitability pathways, resulting in distinct physiological influence of rs1006737 risk allele in individuals with BD relative to healthy carriers (Soeiro-de-Souza et al. 2017). Further investigation is warranted to develop a comprehensive understanding of the precise mechanism of rs1006737 in relation to functional connectivity, particularly how youth with BD are different in this regard.

### Limitations

This study should be interpreted in light of several limitations. First, this is an observational and cross-sectional study and therefore precludes any inferences of causation or directionality. Second, despite the relatively large neuroimaging sample, we were underpowered to examine dose-dependent effects of *CACNA1C* rs1006737 (AA vs. AG vs.GG) or to subgroup BD according to features such as BD subtype, symptomatic status, comorbidity, and/or treatment. Similarly, there was only one significant post-hoc pairwise contrast for the ACC seed, which may also relate to sample size. Third, we did not examine whether rs1006737 is associated with peripheral or brain expression of *CACNA1C* gene, which could provide valuable insights into the potential mechanisms underlying the observed findings. Fourth, the BD individuals were recruited across diagnostic criteria and can be viewed as a heterogeneous clinical sample. While this may have reduced signal detection, rs1006737 was identified in similarly heterogeneous samples, and we opted for the current approach to maximize ecological validity.

## Conclusion

In summary, the current study found differential associations of the *CACNA1C* rs1006737 A allele with rsFC in youth with BD as compared to HC youth, within brain regions implicated in emotion regulation, reward, and executive processes. Future studies including task-based analysis, such as facial affective processing tasks and executive function tasks, may facilitate a deeper understanding of brain connectivity within these regions in relation to *CACNA1C* rs1006747 genotypes. Similarly, integration of *CACNA1C*-related peripheral biomarkers is warranted to generate insights regarding biological mechanisms underlying current findings.

## Supplementary Information

**Additional file 1. Supplementary Materials**.

## Data Availability

The datasets used and/or analyzed during the current study are available from the corresponding author on reasonable request. The data are not publicly available due to privacy or ethical restrictions.

## Abbreviations

ACC: Anterior cingulate cortex
BD: Bipolar disorder
GWAS: Genome-wide association studies
HC: Healthy controls
OFC: Orbitofrontal cortex
rsFC: Resting-state functional connectivity
ROI: Regions of interest
SNP: Single nucleotide polymorphism
