# Supplementary material for: The association of genetic variation in CACNA1C with resting-state functional connectivity in youth bipolar disorder
Source: Int J Bipolar Disord. 2023 Jan 13;11:3. doi: 10.1186/s40345-022-00281-5 (PMC9839925; doi:10.1186/s40345-022-00281-5)
Supplement: Supplementary file 1 — Additional file 1. Supplementary Materials. [file 40345_2022_281_MOESM1_ESM.docx]

**Additional file**

*Clinical methods*

Comorbid diagnoses and clinical characteristics (e.g., psychosis, psychotropic and psychosocial treatment history) were collected during the K-SADS-interview. Age of BD onset was defined as the age at which the individual first experienced an episode of mania or hypomania according to DSM-IV, or when study criteria for BD-NOS were met. The Family History Screen was conducted to determine the family [psychiatric history](https://www-sciencedirect-com.myaccess.library.utoronto.ca/topics/medicine-and-dentistry/psychiatric-history) of all first- and second-degree relatives [(Weissman et al. 2000)](https://paperpile.com/c/VZo6gK/syVo). Information regarding lifetime history of sexual and/or physical abuse was obtained via the K-SADS-PL post-traumatic stress disorder screening questions. “Any Anxiety Disorders” included generalized anxiety disorder, separation anxiety disorder, agoraphobia, and anxiety disorder not otherwise specified. SUD included alcohol or drug abuse or dependence. Lifetime nicotine use, also ascertained via the K-SADS-PL, was computed as a “yes” or “no” variable. Socioeconomic status was calculated using the Hollingshead Four-Factor Index [(Hollingshead 1975)](https://paperpile.com/c/VZo6gK/wiAw). Participants’ global functioning over the current period (past month), most severe past, and highest level in the past year were measured by the Children’s Global Assessment Scale (CGAS) [(Shaffer et al. 1983)](https://paperpile.com/c/VZo6gK/FuoT).

*Saliva and DNA extraction*

Eating, drinking, smoking, and chewing gum were avoided 30 minutes prior to saliva collection. A saliva sample (~2 mL) was collected from each subject in an Oragene OG-500 DNA kit (DNA Genotek, Ottawa, ON). DNA extraction was performed in the Neurogenetics Laboratory at the Centre for Addiction and Mental Health (Toronto, Canada) on a chemagen MSM I DNA extractor (Perkin-Elmer, Waltham, MA) as per manufacturer’s instructions. The extracted DNA was quantified using Nanodrop 8000 spectrophotometer (ThermoFisher Scientific, Waltham, MA) and diluted to 20 ng/µL for use in downstream genotyping applications.

*Collection of Study Data*

Study data were collected and managed using REDCap electronic data capture tools hosted at Sunnybrook Health Sciences Centre and later at the Centre for Addiction and Mental Health. REDCap (Research Electronic Data Capture) is a secure, web-based software platform designed to support data capture for research studies, providing 1) an intuitive interface for validated data capture; 2) audit trails for tracking data manipulation and export procedures; 3) automated export procedures for seamless data downloads to common statistical packages; and 4) procedures for data integration and interoperability with external sources [(Harris et al. 2019; Harris et al. 2009)](https://paperpile.com/c/VZo6gK/AQUT+Agpd).

[Harris PA, Taylor R, Minor BL, Elliott V, Fernandez M, O’Neal L, et al. The REDCap consortium: Building an international community of software platform partners. J. Biomed. Inform. Elsevier BV; 2019 Jul;95(103208):103208.](http://paperpile.com/b/VZo6gK/Agpd)

[Harris PA, Taylor R, Thielke R, Payne J, Gonzalez N, Conde JG. Research electronic data capture (REDCap)--a metadata-driven methodology and workflow process for providing translational research informatics support. J. Biomed. Inform. Elsevier BV; 2009 Apr;42(2):377–81.](http://paperpile.com/b/VZo6gK/AQUT)

[Hollingshead AB. Four factor index of social status. Yale Univeristy. New Haven, CT; 1975; Available from:](http://paperpile.com/b/VZo6gK/wiAw) <https://sociology.yale.edu/sites/default/files/files/yjs_fall_2011.pdf#page=21>

[Shaffer D, Gould MS, Brasic J, Ambrosini P, Fisher P, Bird H, et al. A children’s global assessment scale (CGAS). Arch. Gen. Psychiatry. 1983 Nov;40(11):1228–31.](http://paperpile.com/b/VZo6gK/FuoT)

[Weissman MM, Wickramaratne P, Adams P, Wolk S, Verdeli H, Olfson M. Brief screening for family psychiatric history: the family history screen. Arch. Gen. Psychiatry. American Medical Association (AMA); 2000 Jul;57(7):675–82.](http://paperpile.com/b/VZo6gK/syVo)
